# Improving Cardiovascular Health through Nudging Healthier Food Choices: A Systematic Review

**DOI:** 10.3390/nu11102520

**Published:** 2019-10-18

**Authors:** Christine Tørris, Hilde Mobekk

**Affiliations:** 1 Department of Nursing and Health Promotion, Faculty of Health Sciences, Oslo Metropolitan University, 0130 Oslo, Norway; 2 Department of Behavioural Sciences, Faculty of Health Sciences, Oslo Metropolitan University, 0130 Oslo, Norway; hmobek@oslomet.no

**Keywords:** food choice, eating behavior, healthy food, nudging, choice architecture, obesity, cardiovascular health, diabetes, public health, dietary habits

## Abstract

Obesity and metabolic syndrome are considered major public health problems, and their negative impact on cardiovascular disease (CVD) and diabetes mellitus type 2 (DM2) is profound. Targeting modifiable risk factors such as dietary habits is therefore of great importance. Many of today’s health challenges with overweight and obesity may have behavioral roots, and traditional methods such as regulations and campaigns are often insufficient to improve dietary choices. Nudging or choice architecture might be a viable tool to influence people’s everyday choices and behaviors to better outcomes. This paper reviews the current state of the rapidly expanding number of experimental field studies that investigate the effects/associations of nudging on healthy food choices. A systematic literature search was conducted in PubMed, where 142 citations were identified. Based on selection criteria, six randomized controlled trials and 15 non-randomized controlled trials were ultimately included. The results of this systematic review show that many of the studies included traffic-light labeling, which may be a promising strategy. The reviewed findings, however, also highlight the challenges that confront experimental studies examining the impact of nudging on diet.

## 1. Introduction

Metabolic syndrome (MetS) affects public health, and has been associated with a doubling of cardiovascular disease (CVD) risk as well as a five-fold increased risk of diabetes mellitus type 2 (DM2) [1]. To be diagnosed with MetS, three of five risk factors (abdominal obesity, elevated triglycerides, reduced HDL cholesterol, elevated blood pressure, and elevated fasting glucose) have to be found [2]. Obesity seems to be a driver of MetS, and the highest prevalence of MetS is found in obese populations [3]. The syndrome affects public health, as it increases the risk of morbidity and mortality [4]. Studies from most countries indicate that 20–30% of the adult population can be characterized as having MetS, and the prevalence seems to increase with age [3]. In addition, both parity and increased numbers of children have been associated with higher rates of MetS in women [5]. In addition to genetic predisposition, the initiation of MetS is influenced by environmental factors, such as a sedentary lifestyle together with a diet containing excess calories [6,7]. Targeting modifiable risk factors such as dietary habits is therefore of great importance to reduce the number of morbidity and mortality.

Throughout the past decades research in behavioral sciences has revealed that human behavior and decision-making is boundedly rational, and as a result, people make suboptimal, often self-destructive decisions [8]. Therefore, many of today’s health challenges, such as overweight and obesity, have behavioral roots. These unhealthy habits contribute to the development of long-term conditions such as diabetes and cardiovascular diseases. A habit becomes established by repetition and routine [9]. Even though most people are aware that their habits or lifestyle are unhealthy, especially in the long run, they are not able to change their behavior since it is habitual and influenced by the environment where the choices are made [10]. According to Marteau, Hollands, and Fletcher, 2012 [9], a great part of human behavior is automatic and cued by stimuli in the environment, which in turn results in actions unaccompanied by conscious reflection. Vecchio et al., 2019 [11] also argue that food choices are more likely to fall into the category of fast and unconscious decisions. In addition, as stated by Scott and Loewenstein, 2008 [12], “the benefits of eating are immediate and tangible, whereas the benefits of dieting are delayed and intangible” (p. 3819). 

To improve health outcomes nudging or choice architecture (a related term) [13] might be a viable tool to influence people’s everyday choices and behaviors to better outcomes. In their popular book “Nudge: Improving Decisions About Health, Wealth and Happiness” (2008) [8], Richard Thaler and Cass Sunstein suggest that rearranging the contexts (i.e., physical, social, and psychological) where decisions are made may “nudge” or sway people toward preferred options rather than obstructing or imposing behavior. Nudging does not rely on effortful processes but rather on many decisions that are made automatically and unconsciously [14]. Nudging implies an analytical and experimental approach to changing behavior by integrating insights about human behavior and its boundaries, biases, and habits into the choice architecture [15]. There has been an expansion of research regarding how the environment in which our decisions are made influences us. Findings from this research show that behavioral and contextual interventions based on nudging strategies are promising for promoting healthy eating. Hummel et al., 2019 [16] note that nudges are not just a theoretical concept anymore, but something that might affect citizens in many countries through their increased influence in the political decision-making process.

Several systematic reviews have suggested that nudging may be effective in increasing the consumption of healthy food and decreasing the consumption of unhealthy food: Broers et al., 2017 [17] (nudging to encourage people to select more fruit and vegetables), Arno et al., 2016 [18] (nudge strategies and changing adults’ dietary choices to healthier ones), Wilson et al., 2016 [19], (nudging and its influence on adults food and beverage choices) and Bucher et al., 2016 [20] (the effect of positional changes of food placement on food choice). These reviews were based on searches conducted in 2014–2015. One of the reviews reveals that overall nudge strategies increased healthy nutritional choices by 15%. However, laboratory studies accounted for 48% of the included studies [18]. Both lab and field studies were included in the other reviews as well [17,18,19]. Recently, Vecchio et al., 2019 [11] did a systematic review of the literature (2016–2018) where they investigated evidence of nudging approaches to increase healthy food choices. The results showed that more than 80% (21 of the 26 included studies) of the reviewed research reported positive outcomes [11]. Most of the studies were conducted in the field. However, none of the nudge types were considered more effective than others. The review by Vecchio et al., 2019 [11] was based on search conducted in Scopus and Web of Science. In addition, Vecchio et al., 2019 [11] focused exclusively on changes primarily related to choice architecture as defined by Münscher, Vetter, and Schuerle, 2016 [21]. This involved studies that mainly alter the decision structure and the physical environment and excluded interventions such as product labeling, sizing, and functional design. The field is rapidly increasing and to provide further insight and to foster the replication and scalability of empirical studies we wanted to do a search in PubMed based on the framework of Hollands et al., 2013 [13] and further developed by Al-Khudairy et al., 2019 [22]. The use of PubMed adds several unique and relevant references to the review, which were not found/included in the paper by Vecchio et al., 2019 [11]. Therefore, we wanted to contribute to the current literature on the topic by conducting searches in PubMed.

To better understand how nudges may be implemented in real-world settings, this review primarily aims to examine experimental field studies, investigating effects/associations of nudging on healthy food choices, and if there are specific nudges that are more effective than others. We included studies involving actual food choices that can have a real impact on food selection and/or actual food consumption versus perception or choice intentions. This gave further guidance and understanding of how to best implement nudges in a real-world setting. The research question was explored by reviewing experimental field studies conducted on humans, and reporting nudging or eating behavior as being related to healthy food choices.

## 2. Materials and Methods

In order to identify published studies examining nudging and/or the related term choice architecture [13], versus possible effects/associations on healthy food choice in humans, a literature search was performed in PubMed. The combined search terms were (1) *nudge* and *food choice,* (2) *choice architecture* and *food,* and (3) *nudging* and *healthy food*. 

**Figure 1:**
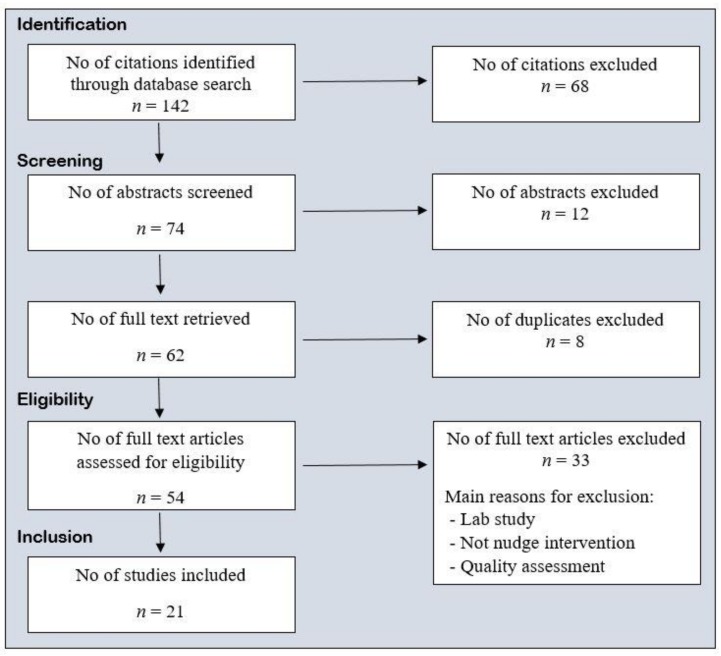
Flow of studies through the different phases of this systematic review.

According to Hollands et al., 2013 [13], choice architecture involves altering small-scale physical and social environments, or microenvironments such as restaurants, cafeterias, lunchrooms, and shops, to cue healthier behavior. This is a more context specific definition than the definition stated by Thaler and Sunstein in “Nudge: Improving Decisions About Health, Wealth And Happiness” in 2008 [23]. The last search was performed in July 2019. Potential abstracts and full-text articles were screened before removing duplicates. Both randomized control trials (RCTs) and non RCTs (published field studies) written in English were included. Animal and lab studies were excluded. A summary of the selection criteria (participants, interventions, comparators, and outcomes) were considered according to PICOS strategy, and is provided in (Table 1). The procedure for the review was carried out in accordance with the PRISMA statement for review reporting [24], and a protocol of the study selection was made. The study selection, which took place during January 2019 to July 2019, was conducted independently by CT and HM. The studies included in the review were assessed using a ten items list (resulting in 11 points), based on the checklist proposed by [25], a checklist designed for both randomized and nonrandomized studies. In the checklist, an overall quality assessment score was calculated. This was done separately by CT and HM, and then discussed before inclusion. The studies with six points or more were included in the study. The selection process is illustrated via a flow diagram (Figure 1).

The majority of studies regarding nudging and/or choice architecture consist of multiple nudges and/or interventions. This makes it a challenge to map the different nudges and to evaluate their effects. However, Hollands et al., 2013 [13] have developed a provisional typology of choice architecture interventions that enable grouping of studies. The typology is divided into three intervention classes as presented in Table 2.

## 3. Results

The literature search identified 142 citations, of which 31 were found in the first search (*nudge* and *food choice*), 79 in the second search (*choice architecture* and *food*), and 32 in the third search (*nudging* and *healthy food*). After screening 74 studies (i.e., titles and abstracts), 62 full-text articles were retrieved and assessed for eligibility. After removing duplicates and those not making it through quality assessment, 21 studies were included in this review. The included studies comprise of six RCTs and 15 non RCTs. A matrix was designed to get an overview over all the included articles. The articles were structured according to reference, participants/site, and results. The study characteristics are provided in Table 3, and the nudges/interventions are presented in Table 4, Table 5, and Table 6.

### 3.1. Randomized Control Trials

Anzman-Frasca et al., 2018 [26] investigated whether placemats affected children’s meal selection and intake in the US. Fifty-eight families with 4-to-8-year-old children were randomized to return to a quick-service restaurant during an intervention or control period (*n* = 28 intervention, 30 control). The participants were blinded to group assignment. The intervention group received placemats featuring two healthy “Kids’ Meals of the Day” upon the restaurant entry, to nudge children toward healthy options. Forty-eight families had looked at the placemat before ordering (*n* = 18 intervention, 30 control). After the families finished dining, researchers recorded children’s orders and collected leftovers for quantifying dietary intake via weighed plate waste. Families who were exposed to the study placemats ordered more healthy food compared to controls. However, there were no significant differences in dietary intake when comparing the intervention versus control groups overall. Nevertheless, children who ordered one of the promoted healthy entrées consumed less saturated fat across the total meal compared to those who did not (*p* = 0.04).

Cohen et al., 2015 [27] investigated the short- and long-term effects of chef-enhanced meals and extended exposure to choice architecture on healthier school food selection and consumption. In this seven months school-based RCT, children aged 8–16 years in urban, low-income school districts were included (intent-to-treat analysis). Firstly, fourteen schools were randomized to chef (*n* = 4) or control (*n* = 10) for five months. Then, the chef schools were further randomized to chef (*n* = 2) or chef + smart café (*n* = 2), and the control schools were further randomized to smart cafe (*n* = 4) or control (*n* = 6). In the smart café, vegetables were offered at the beginning of the lunch line. Fruits were placed in attractive containers, or next to the cash registers. Signage and images promoting fruits and vegetables were prominently displayed. White milk selection was placed in front of sugar-sweetened milk (e.g., chocolate milk). All the modifications were simultaneously present and applied daily by existing food service staff. School food selection was recorded, and food consumption was measured using plate waste methods. The study revealed no association between the smart café intervention alone and food consumption. Fruit selection increased in the chef (OR, 3.08; 95% CI, 2.23–4.25), smart café (OR, 1.45; 95% CI, 1.13–1.87), and chef plus smart café (OR, 3.10; 95% CI, 2.26–4.25) schools compared with the control schools. Vegetable selection increased in the chef (OR, 2.54; 95% CI, 1.83–3.54), smart café (OR, 1.91; 95% CI, 1.46–2.50), and chef plus smart café schools (OR, 7.38, 95% CI, 5.26–10.35) compared with the control schools.

Greene et al., 2017 [28] conducted a 9 week cluster RCT, to evaluate the impact of fruit-promoting Smarter Lunchroom interventions on middle school students’ selection and consumption of fruits. Ten middle schools (Grades 5–8) were recruited and randomized into a fruit intervention (*n* = 4), vegetable (*n* = 3) or control group (*n* = 3). However, the paper only focuses on the fruit intervention and control groups. The fruit intervention group made changes to the convenience, visibility, and attractiveness of fruit in their lunchrooms for a period of 6 weeks. The control group made no changes. The selection and plate waste data were assessed. Fruit selection increased overall by 36% (*p* < 0.001), and fruit consumption increased overall by 23% (*p* < 0.017) in the fruit intervention group, compared to controls.

Hollands et al., 2018 [29] examined the impact on energy purchased of reduced portion sizes in six worksite cafeterias, in a stepped wedge randomized controlled pilot trial. Each site was randomized to a date of implementation. The intervention comprised reducing the portion sizes by at least 10% (by volume without changing energy density) of specified food items (main meals, sides, desserts, cakes). The primary outcome was total energy (kcal) purchased per day from intervention categories. There was found no significant change when data from all six sites were pooled. However, borderline associations were observed at two sites.

Vasiljevic et al., 2018 [30] investigated the potential impact of calorie labeling on energy purchased in six worksite cafeterias in a stepped wedge RCT. The intervention comprised labeling cafeteria products with their calorie content in the same font style and size as for price. The primary outcome was the total energy (kcal) purchased from intervention items in each cafeteria each day. No overall effect of the intervention was revealed, however, a 6.6% reduction (95% CI −12.9 to −0.3, *p* = 0.044) in energy purchased in the day following the introduction of calorie labeling was found in one site. However, the effect diminished over time. No changes in energy purchased were revealed in the remaining five sites.

Velema et al., 2018 [31] examined the effects of a healthy worksite cafeteria (“worksite cafeteria 2.0” (WC 2.0)) intervention on Dutch employees’ purchase behavior over a 12 weeks period. The intervention consisted of fourteen strategies based on nudging and social marketing theories, involving product, price, placement, and promotion (simultaneously executed), to encourage the employees to make healthier food choices during their daily worksite cafeteria visits. The primary outcome were sales data of selected foods. Higher numbers of healthier (“better choice”) products were sold in the intervention group, compared to control three of seven product groups (healthier sandwiches, healthier cheese, and fruit). The increased sales of these healthier meal options were constant throughout the 12 weeks intervention period.

### 3.2. Nonrandomized Control Trials

Cole et al., 2018 [32] investigated the impact of a dining facility intervention on patron diet quality and meal satisfaction in a nonrandomized, controlled time series study. US Army active duty soldiers were included in the intervention consisting of food placements and nutrition labeling to influence food choice (in addition to new food recipes and revised menus). The primary outcomes of the study were change in dietary intake and diet quality scores (Healthy Eating Index 2010 scores). The intervention resulted in higher diet quality scores compared to controls, in addition to increased consumption of citrus, melon fruits, red and orange vegetables, yoghurt, legumes, and whole grains. In addition, oils and solid fat consumption were decreased.

Hubbard et al., 2015 [33] investigated whether a smarter lunchroom would increase the selection and consumption of fruits, vegetables, and whole grains. The 3 months intervention took place at a residential school, where students (*n* = 43, 11–22 years) with intellectual and developmental disabilities were included. The intervention included: (i) prompting by ‘celebrity servers’, (ii) the creation of fruit and vegetable-inspired artwork for the dining hall, (iii) classroom-based taste-testing activities, and (iv) logo naming and branding activities. Selection and plate waste of foods at lunch were assessed. Smarter lunchroom increased selection (whole grains) and consumption (whole grains, fruit) of healthy food, and decreased selection and consumption of unhealthy food (refined grains).

Kroese et al., 2016 [34] investigated whether repositioning of food products could promote healthy food choices among travelers at a train station. Three snack shops were included: (1) repositioning healthy products, (2) repositioning together with an explanatory sign, and (3) control. More healthy products, but not fewer unhealthy, were sold in both nudge conditions, compared to control.

In a 9 months longitudinal study, Levy et al., 2012 [35] investigated whether a two-phase point-of-purchase intervention improved food choices across racial and socioeconomic (job type) groups. The participants were employees (*n* = 4642) of a large hospital in Boston, MA, US, and regular cafeteria patrons. In the first phase, a traffic-light labeling system was introduced to encourage the patrons to purchase healthy items (labeled green) and avoid unhealthy items (labeled red). In the second phase, certain cafeteria items were rearranged, making green-labeled items more accessible and the red-labeled items less accessible. The main outcome measures were proportion of green or red labeled items purchased. Labeling decreased the red item purchases and increased green purchases. The intervention effects were similar across all race/ethnicity and job types.

Lowe et al., 2010 [36] investigated if environmental changes and pricing incentives would influence employees’ lunch choices. The included participants (*n* = 96, BMI = 29.7 ± 6.0 kg/m^2^) who regularly ate lunch at their workplace cafeteria, were randomly assigned into one of two intervention groups: (1) Environmental change (low-energy-dens foods and food content labeling) or (2) Environmental change, education and pricing incentives. Food intake and energy intake was assessed with scan card technology coupled with computerized cafeteria cash registers. No difference in total energy intake were revealed between the groups over the study period. However, significant changes in energy intake were observed across the groups from baseline to the intervention period, with an increase in the percentage of energy from carbohydrates and a decrease of energy from fat. 

Nikolaou et al., 2014 [37] investigated whether calorie information would help young adults to avoid weight gain, in an interrupted time-series study. Students in full-time education reported weight changes over 36 weeks in two year-groups, each of 120 young adults. Both groups were similar in age, gender, and ethnicity, and living in fully-catered accommodation. In the first year, the participants were observed without calorie-labeling, apart from a 5 weeks pilot. In the second year, calorie-labeling was introduced at main meals for 30 of the 36 weeks. The study found that calorie-labeling was associated with a 3.5 kg less weight gain.

Olstad et al., 2014 [38] investigated if nudging and economic incentive was associated with increased healthy food purchases. The participants were patrons at a recreational swimming pool. Three additive interventions were introduced: (1) signage/menu labels, (2) signage and taste testing, and (3) both nudges together with 30% price reductions. Each period was 8 days in length. The primary outcome was the change in the proportion of healthy items sold. In the full sample, sales of healthy items did not differ across periods, whereas in the subsample, sales of healthy items increased by 30% when a signage + taste testing intervention was implemented (*p* < 0.01).

Seward et al., 2016 [39] investigated if traffic-light labeling and choice architecture interventions improved dietary choices among students at a northeastern US university. The 7 weeks intervention included traffic-light labeling (red: least nutrient rich; yellow: nutrient neutral; green: most nutrient rich), choice architecture (how choices are presented to consumers), and “healthy-plate” tray stickers. Two cafeterias received all interventions, two received choice architecture only, and two were controls. The sales for 6 weeks before and 7 weeks during interventions were reported and using interrupted time-series analyses, changes in red, yellow, and green items served were measured. No significant changes in items served were revealed when intervention sites were compared with controls.

Thorndike et al., 2014 [40] investigated the effectiveness of traffic-light labeling and choice architecture over 24 months in a longitudinal pre–post cohort follow-up study. In a large hospital cafeteria, food items were labeled green (healthy), yellow (less healthy), or red (unhealthy) and rearranged to make healthy items more accessible. The traffic-light and choice architecture cafeteria intervention resulted in increased sale of healthier items over 2 years (from 41% to 46%). In addition, the sales of unhealthy items decreased from 24% at baseline to 20%.

Thorndike et al., 2012 [41] investigated whether a two-phase labeling and choice architecture intervention would increase sales of healthy food in a large hospital cafeteria. Phase 1 consisted of a 3 months color-coded labeling intervention (red = unhealthy, yellow = less healthy, green=healthy), and Phase 2 added a 3 months choice architecture intervention where visibility and convenience of some green items were included. The outcome was relative changes in 3 months sales from baseline to Phase 1, and from Phase 1 to Phase 2. The color-coded labeling intervention improved sales of healthy items, and was enhanced by a choice architecture intervention.

Van Kleef et al., 2018 [42] investigated the effect of whole wheat bread as a default option in a sandwich choice situation. A pilot survey (*n* = 291) examined the strength of combinations of toppings and bread type, and the main study consisting of a two (bread type) by two (topping type) between-subjects design. The included participants (*n* = 226) were given a free sandwich at a university stand with an unhealthy deep-fried snack (croquette) or a healthy topping. About half of the participants were offered a whole wheat bun unless they asked for white bun, and the other half were offered a white bun unless they asked for a whole wheat bun. Regardless of the topping, the whole wheat bun was the default option in 94% of the participants. When the default of bread offered was white, 80% of the participants chose the default option. The study revealed a strong default effect of bread type.

Van Kleef et al., 2015 [43] investigated the effectiveness of “verbal prompting” as a nudge to increase fruit salad sales in a self-service restaurant during breakfast time. After an initial baseline period, the intervention involved four different prompts suggesting ordering a side dish (i.e., orange juice, fruit salad, pancakes) given by cashiers. The sales of orange juice increased significant during the orange juice verbal prompts intervention periods (35% to 42% of all breakfasts sold) compared to baseline (20%). Similarly, sales of fruit salad (9%) and pancakes (3%) rose to a small but significant extent compared to baseline sales (3% and 1%, respectively).

Van Kleef et al., 2014 [44] investigated whether the shape of bread rolls is able to shift children’s bread choices from white to whole wheat to increase whole grain intake. In a between-subjects experiment conducted at twelve primary schools in the Netherlands, children were exposed to an assortment of white and whole wheat bread rolls, both varying in shape (regular versus fun). Children were free to choose the type and number of bread rolls and toppings to eat during breakfast, and consumption of bread rolls was measured at class level (number of bread rolls before and after breakfast). In addition, children (*n* = 1113) responded to a survey including questions about the breakfast. Results showed that consumption of fun-shaped whole wheat bread rolls almost doubled consumption of whole wheat bread (*p* = 0.001). However, consumption of white bread rolls did not differ according to shape.

Van Kleef et al., 2012 [45] investigated how manipulation of the assortment and shelf layout near the checkout counter could guide the customers to select healthier snacks. The study applied a two-factor experimental design manipulating snack offerings in a hospital canteen. The shelf arrangement (i.e., accessibility) was altered by putting healthy snacks at higher shelves versus lower shelves, and the assortment structure (i.e., availability) was altered by offering an assortment that either included 25% or 75% healthy snacks. Daily sales data were collected for a period of four weeks. The study revealed that assortment structure led to higher sales of healthy snacks.

Vermote et al., 2018 [46] investigated associations between portion size reduction and french fries consumption, in a pre–post real-life experiment. The participants consisted of university students and employees from Belgian, in an on-campus restaurant setting. The intervention consisted of a reduction of the french fries’ portions by 20%, by replacing the usual porcelain bowl served during the baseline week (±200 g) with smaller volume paper bags during the intervention week (±159 g). French fries consumption and plate waste were measured in 2056 consumers at baseline and 2175 consumers at intervention. Total french fries intake decreased by 9.1%, and total plate waste decreased by 66.4%. No differences were found in satiety or caloric intake between baseline and intervention week. The majority (*n* = 24, 86%) of french fries consumers noticed the reduction in portion size during the intervention.

### 3.3. Mapping of the Nudges in the Included Studies

Al-Khudairy et al., 2019 [22] have modified Hollands et al., 2013 [13] typology and included pricing as one nudge/intervention type. Additional, Al-Khudairy et al., 2019 divide the effects in their systematic review on purchasing or on dietary consumption. This is also relevant for this systematic review. We have modified it further by dividing the dietary consumption into short-term (<6 months) and long-term effects for 6 months or longer. The mapping of the nudges in the included studies are presented in Table 4, Table 5 and Table 6.

## 4. Discussion

Nudging or choice architecture interventions aim to improve dietary choices, but empirical evidence to support the effectiveness has been scarce. An important criterion for an intervention to qualify as a nudge is that the targeted audience/participants/customers retain their freedom to make a choice [23]. In this systematic review, we explore whether behavioral interventions restricted to nudging or choice architecture can influence healthier food choices. Further, we wanted to investigate if there are specific nudges that are more effective. We identified 21 papers that both met the inclusion criteria and the quality check. The core focus in the studies, as this review also reveals, is choice architecture. Fourteen studies examined more than one nudge, and of these studies six studies had more than one condition (A and B or A, B, and C). Seven studies examined pure choice architecture.

### 4.1. Primarily Alter Properties of Objects or Stimuli

Labeling is the most frequent used nudge in this review and nineteen of the studies include labeling. Previous studies have shown mixed results and labeling has been debated [47]. Despite the disputes of labeling, several states and municipalities in the US have introduced regulations that mandate calorie labeling on menus and menu boards in restaurants [48]. The results of the study of Downs et al., 2013 [48] did not support the calorie labeling recommendation, but the authors agree that transparency is beneficial. The traffic-light approach has been regarded as the most effective intervention when it comes to labels. This is simple, informative, and people are already familiar with the connotation of the different colors compared to nutrition labels that are considered more complex to understand. The results in this systematic review is in the line with previous research where the study of Vasiljevic et al., 2018 [30] used calorie labeling in six worksite cafeterias, and only one site showed a statistically significant effect, whereas Levy et al., 2012 [35] and Thorndike et al., 2014 [40] used color-coded food labeling, and both studies had an impact on food choices. While the study by Seward et al., 2016 [39] did not demonstrate any effect by using traffic-light labels. Nine studies used presentation but only Cohen et al., 2015 [27] used presentation alone as a nudge, which was their first condition (A). This had an effect on short-term dietary consumption. Sizing can be both reducing plates and portions, and it is an easy way to reduce consumption and energy of food, but only three studies included reduction of portion size. These studies showed mixed results. The study by Hollands et al., 2018 [29] did not show any significant effect. The effect, where significant, might be due to what Geier et al., 2006 [49] proposed to be unit bias. Unit bias refers to that one single portion, within a reasonable range of size, is seen as a unit to consume. A side effect of a sizing nudge can be reduced food waste as found in Kallbekken and Sælen, 2013 [50]. Hollands et al., 2015 [51] found a clear relation between people being exposed to larger sized portions, such as individual units or tableware, and consuming larger quantities of food compared with people exposed to smaller sizes or units. Pricing is a well-used marketing strategy but only three studies used pricing as a nudge, and always in combination with other nudges. Pricing is also a traditional financial incentive, and one can discuss whether that counts as a nudge according to the original definition by Thaler and Sunstein, 2008 [23].

### 4.2. Primarily Alter Placement of Objects or Stimuli

Availability refers to how accessible an object or stimuli are, and rearranging the positions of food is a common nudge, for instance, in food stores. Seven studies used an availability nudge. Placing healthy food near the cash register increase the sale of these food because people are prone to pick up something in the “last-minute” as done in the study by Van Kleef et al., 2012 [45]. However, in the study of Kroese et al., 2015 [34] the customers did not buy fewer unhealthy products. In the study done by Lowe et al., 2010 [36] the dietary intake improved but there was no difference in the total energy intake. Replacing unhealthy food with healthier options does not necessarily mean a reduction in total energy intake, which is needed to reduce weight. Proximity nudges were used in 11 studies and the majority of these studies made healthier options easier to choose. Van Kleef et al., 2012 [45] used both availability and proximity which resulted in a significant effect on the purchasing of healthier food. Sometimes, availability and proximity can be overlapping nudges, depending on how it is interpreted. 

### 4.3. Alter Both Properties and Placement of Objects or Stimuli

Priming is, according to Marteau et al. (2012) [9], a promising strategy to reduce consumption, but there are few studies so far to evaluate the effectiveness. Priming is used in only four studies, and alters both properties and placement of objects or stimuli. These are incidental cues in the environment that influences us. The effects were mixed, but the studies also used multiple nudges. Prompting refers to labels, signage, or other elements such as placemats used in Anzman-Frasca et al., 2018 [26], but in this study it did not have any effect on dietary consumption. However, in the study by Kroese et al., 2015 [34], an additional sign did not seem to have any added benefit to healthy choice. A prompt can also be verbal, as done in the study by Van Kleef et al., 2015 [43] which resulted in increased fruit purchases.

The results of this systematic review show that the majority of the studies include traffic-light labeling, and that may be a promising strategy. The results suggest that the majority of the interventions were effective. Thirteen studies measured effects on purchasing healthier food, and nine of them, plus one site in the study of Vasiljevic et al., 2018 [30], showed significant effects. Ten studies measured healthier dietary consumption. Only four of the studies measured long-term effect, and with varying results. The dietary intake did not necessarily in all studies affect positive total energy balance that is needed to challenge overweight or obesity. Few of the studies examined how participants altered their purchasing behavior or the side effects of the intervention, for instance whether healthier lunches resulted in increased calorie intake later in the day. Lowe et al., 2010 [36] did examine the effect pre- and post-intervention but argued that the results could be a Hawthorne effect. Therefore, to gain further insight into whether a nudge intervention has an effect, it is important to record and examine changes in individual choices before and after a nudge intervention.

In most of the field experiments, the participants were not aware that they were part of an experiment. This limited social influence regarding desirable behavior and observer reactivity [52]. The field nature in the majority of the studies (e.g., workplaces, cafeterias, and lunchrooms) represents an important strength of the research within the nudging field. It is a challenge to obtain experimental control and replication of studies since real-life settings are complex. However, field studies, together with lab experiments, are important for building convincing evidence for interventions. The lack of control groups and small sample sizes are limitations that diverge from more traditional healthcare research where the gold standard is randomized control studies. The lack of blinding and randomization might be necessary to enable a realistic choice setting in natural field-experiments. This systematic review included six RCT studies compared to fifteen non RCT. Nudge interventions are context specific and the difference in gender, age, ethnicity, and education limit generalizability from one study to another.

### 4.4. Implications for Research and Practice

Despite the tremendous interest and studies on nudging and choice architecture regarding public health, it is still a relatively new and under-explored field. Many of the studies reviewed during this systematic review lacked definitional and conceptual clarity that might lead to poor methodology and unclear effects. A benefit of nudging is that it does not requires any additional actions for the individual or the targeted audience. Furthermore, the interventions are usually no cost or low cost, and that makes it easier to implement. Another important issue is that our environment, whether we like it or not, influences our behavior. Then it makes sense to alter the environment in a way that we make better choices that will be beneficial in the long run [10]. Many of the nudging or choice architecture interventions as shown in this review have potential to be scaled up to a higher population level. Nudging, though, is not necessarily a salvaging concept as viewed across many disciplines [16]. Therefore, it is important to have a continuous scientific evaluation, since it affects many citizens throughout the world and interventions might not have any effect, a perverse effect, or even backfire.

### 4.5. Strengths and Limitations of This Study

This systematic review was executed independently by both authors to minimize errors and bias. When there was discrepancy regarding a study, the study was thoroughly discussed based on the inclusion criteria and the quality check list. We prepared a checklist for measuring study quality based on the Downs and Black validated checklist from 1998 [19]. Consumption of sugar-sweetened beverages (SSB) is cited as a major contributor to the obesity epidemic [53], but research regarding SSBs only was not included in this review, since the objective of this paper was food. The data source in this systematic review was limited to PubMed. This might have had an impact on the number of included studies. Another limitation that is relevant to this systematic review is that nudging or choice architecture studies usually examine more than one intervention at a time, and this makes it difficult to draw conclusions on the effects of each single nudge.

Nudging and/or choice architecture alone will not solve the worldwide health challenges caused by poor health choices. Although behavioral economics can provide great benefits if appropriately used, it is still necessary with an underlying political fundament [47]. In other words, nudge interventions should complement, rather than substitute for, more forceful and traditional policies (e.g., Liu et al., 2014 [54]; Vecchio et al., 2019 [11]). A threat raised by Marteau et al., 2012 [9] is posed to economics that are built on excessive consumption. Successful behavioral-based efforts to prevent diseases caused by overweight and obesity would reduce the consumption of food, and that again would have a great impact on a lot of stakeholders. Nudging again is only one part of behavioral economics, and should be regarded as one more tool to prevent noncommunicable diseases. The results of this systematic review, as in the review by Hummel et al., 2019 [16], indicate that the evidence is rare. In this review, for instance, the study of Olstad et al., 2014 [38] did not result in any effect in the full sample population. They emphasize that nudging is a subtle technique and that choices are influenced by other factors such as marketing and food preferences. Another issue is the publication bias—that studies without any significant effects are not published [16], so the studies by Holland et al., 2018 [29], Olstad et al., 2014 [38], and Seward et al., 2016 are rare.

Moving forward, nudging and choice architecture would benefit from a better conceptual clarity and a more systematic approach. There are no “quick fix” or “magic bullets” when it comes to changing people’s behavior. Additionally, even though it might be challenging, nudging interventions should be compared to more traditional interventions such as information campaigns and educational strategies. Future research should have longer study durations such as Thorndike et al., 2014 [40], and include a follow up study to see if the nudge or choice architecture have a long-term impact. It also adds value to include pictures of the nudges, as some studies do, to easier understand the actual intervention and to evaluate the study, and not least to easier access replication. In addition, to cope with the challenges regarding overweight and obesity other interventions might be needed. Modern society fuels the prevalence of unhealthy diets by increasing the accessibility and availability of unhealthy food with, for instance, conveniently located fast-food restaurants [19].

## 5. Conclusions

The results of this systematic review show that the effect sizes are very diverse and also low. Many of the studies included traffic-light labeling that might be a promising strategy. Moreover, this study also highlights the challenges that must be addressed when experimental studies concerning nudging are conducted. According to Marteau et al. (2012) [9], we live in an environment that is exerting strong negative impacts on our health, and it remains to be seen whether we can turn the negative trend. Retailers, cafes, restaurants, cafeterias, and canteens all play an important role in shaping our food habits because they decide which products to sell [55]. Using traffic-labeling to guide us, reducing portion sizes in restaurants and cafes, smaller plates in lunch buffets, healthier options in the workplace canteen, healthier school meals, and some “nudged” choices each day with less calories and healthier nutrition, small cumulative consequences or the “peanuts effect” [56] might have an effect on our health in the long run.

## Figures and Tables

**Table 1:** Inclusion and exclusion criteria.

PICOS	Inclusion Criteria	Exclusion Criteria
Population	Humans	Animal studies
Intervention	Nudging interventions aimed at increasing healthy food choice	Lab studies Not food
Comparison		
Outcome	Food choice/consumption	Studies that do not report food choice/intake as primary outcome
Study design	Randomized and nonrandomized controlled trials (quasi-experimental study)	Abstracts and protocols

**Table 2:** Nudging and choice architecture typology from Al-Khudairy et al., 2019 [22] and Hollands et al., 2013 [13].

Intervention Class	Intervention Type
Primarily alter properties of objects or stimuli	Ambience—alter aesthetic or atmospheric aspects of the surrounding environment
	Functional design—design or adapt equipment or function of the environment
	Labeling—apply labeling or endorsement information to product or at point-of-choice
	Presentation—alter sensory qualities or visual design of the product
	Sizing—change size s of the product
	Pricing—change price of the product
Primarily alter placement of objects or stimuli	Availability—add behavioral options within a given microenvironment
	Proximity—make behavioral options easier or harder to engage with, requiring reduced or increased effort
Alter both properties and placement of objects or stimuli	Priming—place incidental cues in the environment to influence a non-conscious behavioral response
	Prompting—use nonpersonalized information to promote or raise awareness of a behavior

**Table 3:** Characteristics of included studies.

Reference	Participants/Site	Results
RCTs		
Anzman-Frasca et al., 2018 [26]	58 families with 4–8 year old children, quick-service restaurant	Placemats: ordered more healthy food compared to controls (B = −1.76, 95% CI −3.34, −0.19). No (overall) differences in dietary intake compared to control.
Cohen et al., 2015 [27]	Students 1–8 grade urban, low-income districts, school cafeteria	Fruit and vegetable selection increased in smart cafe, however smart café intervention alone had no effect on consumption.
Greene et al., 2017 [28] 9 week cluster	Ten middle schools (5–8 grade), cafeteria	Overall, fruit selection increased by 36% (*p* < 0.001), and fruit consumption increased by 23% (*p* < 0.017) in the fruit intervention group, compared to controls.
Hollands et al., 2018 [29] stepped wedge	Nine worksite cafeterias	No significant change in daily energy purchase when data from all six sites were pooled.
Vasiljevic et al., 2018 [30]	Six worksite cafeterias	No overall effect in energy purchase. One site 6.6% reduction (95% CI −12.9 to –0.3, *p* = 0.044) in energy purchased, however, the association diminished over time.
Velema et al., 2018 [31]	Employees	Positive effects on purchases for three of seven products
Non RCTs		
Cole et al., 2018 [32]	US Army active duty soldiers, military installation	Intervention associated with increased diet quality and consumption of healthy food.
Hubbard et al., 2015 [33]	Students (n 43) 11–22 years with intellectual and developmental disabilities	Smarter lunchroom increased selection (whole grains) and consumption (whole grains, fruit) of healthy food.
Kroese et al., 2015 [34]	Travelers, train station snack shops	More healthy (but not fewer unhealthy) products were sold in both nudge conditions.
Levy et al., 2012 [35]	Employees who were regular cafeteria patrons (*n* = 4642)	Labeling decreased unhealthy purchases and increased healthy purchases.
Lowe et al., 2010 [36]	Employees, worksite cafeteria	Total energy intake: no difference. Dietary intake improved over study period.
Nikolaou et al., 2014 [37]	120 students, catering	Calorie-labeling associated with a 3.5 kg less weight gain.
Olstad et al., 2014 [38]	Patrons, recreational swimming pool	In the full sample, sales of healthy items did not differ across periods. In the subsample, the sale of healthy items increased by 30% when signage + taste testing was implemented (*p* < 0.01).
Seward et al., 2016 [39]	6 college cafeterias (Harvard University, Cambridge, Massachusetts)	No significant changes (items served) were revealed when intervention sites were compared with controls.
Thorndike et al., 2014 [40]	Cafeteria	The traffic-light and choice architecture cafeteria intervention resulted in increased sale of healthier items over 2 years (from 41% to 46%).
Thorndike et al., 2012 [41]	Hospital cafeteria	A color-coded labeling intervention improved sales of healthy items and was enhanced by a choice architecture intervention.
Van Kleef et al., 2018 [42]	Participants at a Dutch university	Regardless of the topping, when the whole wheat bun was the default option, 94% decided to stick with the default.
Van Kleef et al., 2015 [43]	Customers in self-service restaurant during breakfast	The sales increased significant during the verbal prompts intervention periods compared to baseline.
Van Kleef et al., 2014 [44]	Children (*n* = 1113) primary schools in the Netherlands	Consumption of fun-shaped whole wheat bread rolls almost doubled consumption of whole wheat bread (*p* = 0.001).
Van Kleef et al., 2012 [45]	Students	Assortment structure led to higher sales of healthy snacks.
Vermote et al., 2018 [46]	University students and employees	Total french fries intake decreased by 9.1%, and total plate waste decreased by 66.4%. No differences in satiety or caloric intake (dietary recall) between baseline and intervention week.

RCT: Randomized Control Trials.

**Table 4:** Nudging and choice architecture types and effectiveness examined in the included studies.

Intervention Class	Intervention Type	Anzman-Frasca et al. 2018 [26]	Cohen et al. 2015 [27]			Cole et al. 2018 [32]	Greene et al. 2017 [28]	Hollands et al. 2018 [29]	Hubbard et al. 2014 [33]	Kroese et al. 2015 [34]	Levy et al. 2012 [35]	
			A	B	C						A	B
Primarily alter properties of objects or stimuli	Ambience											
	Functional design											
	Labeling	X		X	X	X	X				X	X
	Presentation		X	X			X		X			
	Sizing							X	X			
	Pricing											
Primarily alter placement of objects or stimuli	Availability					X	X		X			
	Proximity			X	X		X		X	X		X
Alter both properties and placement objects and stimuli	Priming									X		
	Prompting	X							X	X		
Effect	On food choice							N		Y	Y	Y
	On dietary consumption Short-term	N	Y	Y	N	Y	Y		Y			
	On dietary consumption Long-term		N	Y	N	Y						

A, B, and C refer to different conditions in the same study. Effect: Short-term <6 months, and long-term ≥6 months. X: Nudging and choice architecture types. Y: Yes, N: NO.

**Table 5:** Nudging and choice architecture types and effectiveness examined in the included studies.

Intervention Class.	Intervention Type	Lowe et al. 2010 [36]		Nikolaou et al. 2014 [37]	Olstad et al. 2014 [38]			Seward et al. 2016 [39]	Thorndike et al. 2014 [40]		Thorndike et al. 2012 [41]	
		A	B		A	B	C		A	B	A	B
Primarily alter properties of objects or stimuli	Ambience											
	Functional design											
	Labeling	X	X	X	X	X	X	X	X	X	X	X
	Presentation						X			X		X
	Sizing											
	Pricing		X				X					
Primarily alter placement of objects or stimuli	Availability	X	X					X				
	Proximity									X		X
Alter both properties and placement objects and stimuli	Priming					X	X					
	Prompting											
Effect	On food choice	Y	Y		N	N	N	N	Y	Y	Y	Y
	On dietary consumption Short-term											
	On dietary consumption Long-term			Y								

A, B, and C refer to different conditions in the same study. Effect: Short-term <6 months, and long-term ≥6 months. X: Nudging and choice architecture types. Y: Yes, N: NO.

**Table 6:** Nudging and choice architecture types and effectiveness examined in the included studies.

Intervention Class.	Intervention Type	Van Kleef et al. 2018 [42]	Van Kleef et al. 2015 [43]	Van Kleef et al. 2014 [44]	Van Kleef et al. 2012 [45]	Vasiljevic et al. 2018 [30]	Velema et al. 2018 [31]	Vermote et al. 2018 [46]
Primarily alter properties of objects or stimuli	Ambience							
	Functional design							
	Labeling					X		
	Presentation			X			X	
	Sizing						X	X
	Pricing						X	
Primarily alter placement of objects or stimuli	Availability				X		X	
	Proximity	X			X		X	
Alter both properties and placement objects and stimuli	Priming						X	
	Prompting		X					
Effect	On food choice	Y	Y		Y	N/Y	Y	N
	On dietary consumptionShort-term			Y				Y
	On dietary consumptionLong-term							

A, B, and C refer to different conditions in the same study. Effect: Short-term <6 months, and long-term ≥6 months. X: Nudging and choice architecture types. Y: Yes, N: NO.
